# Occurrence of *Escherichia Coli* O157:H7 in lactating cows and dairy farm environment and the antimicrobial susceptibility pattern at Adami Tulu Jido Kombolcha District, Ethiopia

**DOI:** 10.1186/s12917-023-03565-9

**Published:** 2023-01-11

**Authors:** Frehiwot Mesele, Samson Leta, Kebede Amenu, Fufa Abunna

**Affiliations:** 1 Adami Tulu Agricultural Research Center, P.O. Box 35, Ziway, Ethiopia; 2 Addis Ababa University, Collage of Veterinary Medicine and Agriculture, P.O.Box 34, Bishoftu, Oromia Ethiopia

**Keywords:** Antimicrobial susceptibility test, Dairy farm environment, *E. coli* O157:H7, Lactating cows

## Abstract

**Background:**

Food-borne pathogens are the foremost causes of food-borne human illness in the world. *Escherichia coli* O157:H7 (*E. coli* O157:H7) is one of the major food-borne pathogenic bacteria around the world. Though evidence is lacking; especially in developing countries like Ethiopia, the potential health impact of *E. coli* O157:H7 can be high where food production, handling and consumption is often taking place under unhygienic conditions. In Ethiopia, studies reported *E. coli* and *E. coli* O157: H7 from food of animal origin, mainly meat and milk, and also animal surfaces and feces. The objective of the present study was to investigate the occurrence of *E. coli* O157:H7 in raw milk and the dairy production farm environment and further assess the antimicrobial resistance pattern of the bacterium.

**Methods:**

Samples of milk from individual lactating cows’ and dairy farm environmental samples (feces, water and manure) were collected at Adami Tulu Jido Kombolcha district (ATJKD) and analyzed for the presence of *E. coli* O157:H7. Standard microbiological techniques including culture, biochemical testing and serological test were performed to isolate and identify the bacterium. The bacterial isolates were evaluated for antimicrobial susceptibility patterns using disk diffusion method. A questionnaire was used to collect possible factors affecting *E. coli* O157:H7 occurrence.

**Results:**

The overall prevalence of *E. coli* O157:H7 was 4.7% (19/408) (95% CI: 2.6; 6.7). Out of 19 *E. coli* O157:H7 isolates, 4/50, 7/154, 2/50, and 6/154 were from water, milk, manure, and feces samples, respectively. From potential risk factors considered in this study area, floor type, cleaning of pens, milking location and hand washing during the time of milking were significantly associated with the occurrence of *E. coli* O157:H7. The antimicrobial susceptibility pattern indicated varying degrees of resistance. All of the isolates were found to be resistant ampicillin, cephalothin, and rifampin, and 100% susceptibility was observed against the drugs: chloramphenicol, ciprofloxacin, gentamicin, nalidixic acid, kanamycin, and tetracycline. Concerning streptomycin, 63.15% of the isolates were susceptible and 36.8% showed intermediate susceptibility.

**Conclusions:**

The occurrence of multi-drug resistance *E. coli* O157:H7 observed both in lactating cows and in dairy farm environments can sustain a continuous transmission of the bacteria. The occurrence of multidrug-resistant *E. coli* o157:H7could hamper the control and prevention efforts.

**Supplementary Information:**

The online version contains supplementary material available at 10.1186/s12917-023-03565-9.

## Introduction

Foodborne pathogens are prominent causes of foodborne illness with huge public health and economic consequences [1]. They place a heavy burden costing billions of dollars in medical care, social costs, and overall economic and infrastructure effects on countries [2]. Foodborne diseases disproportionately affecting developing countries of the world due to major contributing factors such as overcrowding, poverty, changes in eating habits, mass catering, complex and lengthy food supply procedures with increased international movement, inadequate sanitary conditions, and poor general hygiene practices [3, 4]. The emergence of major foodborne pathogens such as Salmonella and *E. coli* has persisted as a public health concern of foodborne pathogens despite considerable efforts aimed at prevention and control [5]. Around 2 million people die per year due to diseases of foodborne pathogens [6]. Consumption of raw/undercooked meat, vegetables, and fruits are the main sources of infection that leads to public health significance of *E. coli* O157:H7 [7, 8].

*E. coli* O157:H7 are commonly found in farm animals in cattle gut [9, 10]. Contaminated feed and water, the immediate environment of the animal, and cattle feces have been considered the primary source of animal origin of *E. coli* O157:H7 in human infection [9, 11]. *E. coli* O157:H7 can cause from mild cases of diarrhea to severe illness in humans such as hemorrhagic colitis, hemolytic-uremic syndrome (HUS), and thrombotic thrombocytopenic purpura (TTP) leading to death [12, 13]. In Ethiopia, raw milk, minced raw meat [14, 15], cream, creamed fish, vegetables, and poultry and their products are regarded as high-risk commodities in respect of pathogen contents, natural toxins, and other possible contaminants and adulterant [16–18].

The farm environment is the main factor in sustaining a population of viable *E.coli* O157:H7 which can survive in feces, manure, pen surfaces, bedding, flooring, and water [10, 19–23]. Specifically, cattle manure could allow prolonged survival of the bacterium outside the host [24]. Contaminated drinking water may contribute to the dissemination and maintenance of *E. coli O157:H7* on farms [21].

Antimicrobials are used as the therapeutic agents in humans and in animals as therapeutics or prophylaxis.. Inadequate selection and abuse of antimicrobials may lead to resistance in various bacteria and make the treatment of bacterial infections difficult [25]. Antimicrobial resistance in *E. coli* O157:H7 has been reported worldwide [26]. Treatment for *E. coli* infection has been increasingly complicated by the emergence of resistance to most first-line antimicrobial agents [27]; including ampicillin, amoxicillin, ceftriaxone, chloramphenicol, ciprofloxacin, cotrimoxazole and tetracycline [28–30].

In Ethiopia, studies stated that the presence of multi-drug resistance (MDR) in various livestock production systems to diverse antimicrobial drugs. In Ethiopia, the prevalence of MDR ranges from 5.7 to 100% according to Hiko et al*.* [31], Messele et al [32] Shecho et al [33], Abebe et al [34], Haile et al [35], Mohammed et al [16], Dejene et al [36], Haile [37], Tassew [38] and Taye et al [39] from raw meat, and dairy products at Bishoftu, Modjo, Addis Ababa, Haramaya, Tigray testing resistance of different human importance antibiotics including Ampicillin, cephalothin, gentamicin and tetracycline.

In developing countries like Ethiopia, the production of animal source foods often takes place under unhygienic conditions and; consumption of raw products can be common. Due to the unhygienic handling, the foods might be contaminated by feces, manure, and poor quality water in dairy farms with further potential hazards and source of infection for humans [9].. Epidemiology of food-borne pathogens specifically that of *E. coli* O157: H7 is not well studied. But recently, considerable studies have been reported on the occurrence of *E. coli* O157: H7 from food of animal origin mainly raw meat from abattoirs, butcher shops, and restaurants according to these authors [8, 14, 15, 31, 32, 34, 35, 38–45]; Similarly, reports of the occurrence of the pathogen from dairy products from farms, milk vendors and supermarkets are available [15, 34–36, 46–49]. Some studies also covered isolation of the pathogen from feces, water, and contact surfaces such as farms and slaughterhouses [8, 36, 37, 40, 41, 44, 45] in different parts of Ethiopia. The isolation of the pathogen associated with common practices of raw animal products consumption in many circumstances increase the risk of *E*. coli O157:H7 infection. The close contact of humans with cattle feces and manure can be another direct source of infection to humans.

The prevalence estimates ranged from 0.01 to 13.4%. Assefa and Bihon [50] conducted a meta-analysis to estimate the pooled prevalence of *E. coli* and *E. coli* O157:H7 and reported a pooled prevalence of 15% (95% CI = 13–17%) for *E. coli* and 4% (95% CI = 3–5%) for *E. coli* O157:H7. Therefore studies revealed that *E. coli* O157:H7 is an important food-borne pathogen in humans and MDR is a major problem and there is limited information on the occurrence of *E. coli O157:H7* and its antibiotic resistance pattern in lactating cow and dairy farm environment in the present study area. Thus, this study aimed to assess the occurrence of *E. coli* O157:H7 in lactating cows and dairy environments. In addition, risk factors associated with *E. coli* O157:H7 occurrence and antimicrobial resistance profile of the isolates were evaluated.

## Materials and methods

### Study area

Adami Tulu Jido Kombolcha district (ATJK) is found in the mid-Rift Valley at 7° 9′N latitude and 38° 7 ‘E longitude Livestock production is the dominant farming system and crop production is not common. Dairy cattle are mostly reared in small to medium scale dairy operations, in which animals are managed both intensively and extensively. They are often provided with some supplementary diet in addition to the natural pasture and agricultural by-products [51]. But there is no large-scale dairy farm in the area and herd size in the dairy farms ranges 3 to 31 heads of cattle.

### Study design, sampling methods and sample size determination

The study population was lactating dairy cows in ATJK district and comprises exotic, crossbred and local breeds in small and medium scale dairy farms managed under intensive and extensive management conditions. At the animal level, raw milk and feces samples were collected from the teat and rectum of selected dairy cows, respectively. From the dairy farm environment, manure and water samples were also collected. Semi-structured questionnaire was used to interview the farm owners (supplementary file).

A stratified random sampling method was used to sample dairy farms. The farms were categorized based on their herd size into three strata (small-scale < 10 animals), medium-scale (10 to 50 animals) and large-scale (> 50 animals) using the classification made by Megersa et al [52]. Based on the data obtained from the district livestock and fishery agency there are 22 medium, 68 small-scale and no large-scale dairy farm in the district. Then dairy farms and individual lactating cows were selected using simple random sampling method. Random sampling technique was used to recruit farmers and there was no compensation given to them and their participation was on voluntary basis with their full consent.

Sample size was determined by using the formula given by Thrusfield [53] with consideration of 95% confidence interval and 5% precision. The expected prevalence was set at 10.4% based on a previous study conducted by Abebe et al [34] resulting in 144.. Thus, 154 dairy cows in 50 dairy farms were sampled. The total sample size then became 408 (154 milk samples, 154 feces samples, 50 water and 50 manure samples).

### Sample collection

All the samples after collection were tagged by animal ID, date of sampling and sample type. The samples were transported to the Veterinary Microbiology of Laboratory, of the College of Veterinary Medicine and Agriculture of Addis Ababa University using an icebox maintaining a cold chain for microbiological analysis. Upon arrival, the samples were stored in a refrigerator at 4 °C for 24 hours until being processed for isolation as described by Quinn et al. [54].

Milk samples were collected directly from teats by sterile screw topped universal bottle. The fecal samples were collected using a sterile stomacher bag aseptically directly from the rectum and stored in an icebox until analysis (within 24 hours). Milk and feces samples were collected from the same animal. Water samples (10 ml) were collected using sterile capped universal bottles. Pooled manure samples were also collected from the selected dairy farms using sterile stomacher bags from different points including pen, floor surface and dung storage area. Isolation and identification of *E. coli* O157:H7 was made based on the colony morphology in different media, staining characteristics and biochemical properties [55].

### Sample processing

Non-selective pre-enrichment was necessary for the effective recovery of low levels of stressed *E. coli* O157. All sorbitol non-fermenting colonies that reacted with O157 were considered presumptive *E. coli* O157:H7 for further analysis. Thus, enrichment was conducted according to OIE guidelines [56] All enriched samples were cultured on a sterilized Sorbitol MacConkey (SMAC) agar plate, (CM0813, Oxoid Basingstoke, England) and the confirmed pure cultures were transferred to nutrient agar to be used for additional biochemical and serological confirmation as described by Quinn et al. [54]. The non-sorbitol fermenting (NSF) *E. coli* (colorless or pale colonies) was considered as *E. coli* O157: H7 strains whereas pinkish-colored colonies (sorbitol-fermenters) were considered as non-O157: H7 *E. coli* strains. The NSF isolates were again subjected to latex *E. coli* O157: H7 agglutination test for confirmation.

Serological confirmation of *Escherichia* strains possessing the O157 serotype antigen was done using dry spot *E.coli* O157 latex agglutination test according to the manufacturer’s instruction (Oxoid, DR120M).

### Antimicrobial susceptibility testing

Antimicrobial susceptibility tests were performed by standard disc diffusion technique using commercially available antimicrobial disks. Antimicrobial disks containing ampicillin (10 μg), cephalothin (30 μg), ciprofloxacin (5 μg), chloramphenicol (30 μg), gentamicin (10 μg), kanamycin (30 μg), nalidixic acid (30 μg), rifampin (5 μg), streptomycin (10 μg) and tetracycline (30 μg) (HI media, India) were used. Subsequently, the diameter of the inhibition zone created around each disk was measured using a digital caliper. The results were classified as sensitive, intermediate and resistant according to the standard supplied by CLIS [57] and multidrug resistance refers to the resistance of a single isolate against more than two drugs.

### Questionnaire survey

A semi-structured questionnaire was used to collect additional data on demographic characteristics, milking system, milking and hygienic practices (washing of milkers’ hands, milk utensils and udder before milking), farmers’ awareness of cattle and milk-borne zoonoses, transmission routes, sources of farm water, housing management. The questionnaire was pre-tested on five dairy farm owners. The interview was made in local language (Afaan Oromo or Amharic). All 50 farm owners were interviewed through face-to-face conversation.

### Data management and statistical analysis

Data were entered into a Microsoft Excel spreadsheet (Microsoft Corp., Redmond, WA, USA). Descriptive statistics (determination of proportions) were used to summarize the data. The overall prevalence of *E. coli* O157: H7 in milk, feces and environmental samples was estimated using the standard formula. R statistical software Version 3.3.2 [58] was used to analyze the data. Pearson chi-square, Pearson’s Chi-squared test with Yates’ continuity correction and fisher exact tests were used to assess the association of different risk factors with the occurrence of *E. coli* O157:H7. Univariable and multivariable binary logistic regression analysis were performed to quantify crude and adjusted effect of the risk factors on the occurrence of *E. coli* O157: H7. The step AIC function in ‘MASS’ package was used to select the final multivariable logistic regression model using backward variable elimination process. Likelihood ratio test was used to compare models during model selection. *P*-value less than 5% (*P* < 0.05) was considered statistically significant.. In cases of estimating the effect of different risk factors in terms of Odds ratio (OR) with corresponding 95% confidence interval, statistical significance was assumed if the confidence interval did not include one among its value.

## Results

### Occurrence of *E. coli* O157:H7

Out of 408 samples collected and processed, 19 were positive for *E.coli* O157:H7. The prevalence of *E. coli* O157:H7 in lactating cows (milk and feces) and dairy farm environment (water and manure) at ATJK district were found to be 4.7%. (95% CI: 2.6; 6.7). Of these positive cases, the isolation of *E. coli* O157:H7 was the highest in water sample 4(8%), followed by milk samples 7 (4.5%), in manure 2(4%) and 6 (3.9%) in feces as presented in Table 1. Prevalence of *E. coli* O157:H7 was isolated in 11 (7.1%) individual cows with the reference of feces and milk and 14 (28.0%) at the farm level based on all types of samples.

**Table 1:** Occurrence of *E. coli* O157:H7 in different sample type

Sample type	Total sample examined	***E. coli*** isolates	***E. coli*** O157:H7 strains
Milk	154	15 (9.7)	7 (4.5)
Feces	154	16 (10.4)	6 (3.9)
Water	50	6 (12)	4 (8)
Manure	50	5 (10)	2 (4)
Overall	408	42 (10.3)	19 (4.7)

### Univariable analysis of the association of *E. coli* O157:H7 with different risk factors

The effect of potential risk factors on the occurrence of *E. coli* O157:H7 was assessed and from the risk factors considered, cleaning of pens, milking location, use of towels and hand washing during the time of milking had a statistically significant impact on the occurrence of *E. coli* O157:H7 (P < 0.05) using univariable logistic regression analysis. On the contrary, factors such as the breed of the animal, herd size, area, floor type, use of detergent and history of mastitis did not show significant differences (*p* > 0.05) Table 2.

**Table 2:** Univariable logistic regression analysis of *E .coli* O157:H7 occurrence with various risk factors

Risk factors	*E. coli* O157:H7					
	No. examined	No. of positive	X^2^	P-value	Crude OR	95% CI OR
Area						
Rural	120	8	0.61	–	–	–
Urban	269	11		0.306	0.61	0.24–1.60
Breed						
Exotic	101	1	*	–	–	–
Cross	183	13		0.059	7.17	0.93–55.65
Local	105	5		0.155	4.81	0.55–41.89
Cleaning of pens						
No stay overnight	133	13	7.81^a^	–	–	–
Stay overnight	256	6		0.005^**^	4.17	1.61–12.5
Herd size (Farm scale)						
Medium scale	137	3	2.23	–	–	–
Small scale	252	16		0.095	2.90	0.94–12.62
Milking location						
In barn	151	13	5.43^a^	–	–	–
Anywhere	238	6		0.005^**^	3.45	1.32–10.00
Hand wash						
Before and after milking	377	15	*			
Only before milking	12	4		0.000^***^	8.37	2.15–27.49
Floor type						
Earthen	214	8	0.75^a^	–	–	–
Concrete	175	11		0.275	1.68	0.67–4.42
Use of towel						
No use of towel	96	8	*	–	–	–
Before milking	174	9		0.310	0.60	0.22–1.61
Before and after milking	138	2		0.023	0.16	0.03–0.78
Use of detergent						
No	52	2	*	–	–	–
Yes	356	17		0.767	1.25	0.35–8.06
History of mastitis						
No	104	8	*	–	–	–
Yes	304	11		0.096	0.45	0.18–0.19

*OR* Odds Ratio

*** Significant level (*P* < 0.001), **Significant level (*P* < 0.01), *Computed using fisher exact test, − reference

^a^ Pearson’s Chi-squared test with Yates’ continuity correction

### Multivariable analysis of the association of *E. coli* O157:H7 with different risk factors

From potential risk factors considered in this study (Table 3), area, floor type, cleaning of pens, milking location and hand washing during the time of milking were significantly associated (*P* < 0.05) with the occurrence of *E. coli* O157:H7. As shown in Table 3, the odds ratio of *E. coli* O157:H7 occurrence was 9.32 times higher in urban areas than in rural areas. In pens where the feces stay overnight, the odds ratio of *E. coli* O157:H7 occurrence was nearly 50 times higher. Animals that were milked anywhere on the farm had 16.67 times higher risk compared to animals that are milked in a milking barn. Hand washing practice had also a significant impact on the occurrence of *E. coli* O157:H7, the odds ratio of *E. coli* O157:H7 occurrence in farms where hand washing is practiced only before milking was 8.51 times higher when compared with farms where before and after milking hand wash is experienced. Farms with concrete floor were 48.74 times at higher risk when compared with farms with ordinary floor type (earthen floor).

**Table 3:** Multivariable logistic regression analysis of *E. coli* O157:H7 occurrence with various risk factors

Risk factors	Adjusted OR	95% CI OR	*p*-value
Area			
Rural	–	–	–
Urban	9.32	1.79–48.60	0.008**
Cleaning of pens			
No stay overnight	–	–	–
Stay overnight	50	7.69–50.0	0.000***
Milking location			
In barn	–	–	–
Anywhere	16.67	3.03–83.33	0.001**
Hand wash			
Before and after milking	–	–	–
Only before milking	8.51	1.88–38.49	0.005**
Floor type			
Earthen	–	–	–
Concrete	48.74	3.49–680.66	0.004**

*** Significant level (*P* < 0.001), ** Significant level (*P* < 0.01), − reference

### Antimicrobial susceptibility pattern of isolates

**Fig. 1:**
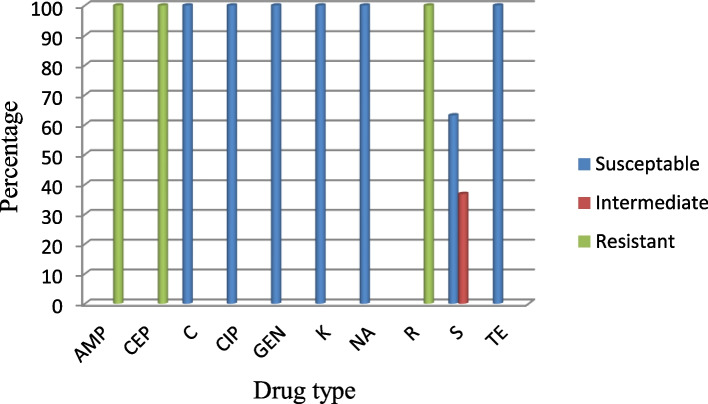
Antimicrobial susceptibility pattern of *E.coli* O157:H7 to ten antimicrobials. Key: AMP: Ampicillin, CEP: Cephalothin C: Chloramphenicol, CIP: Ciprofloxacin, GEN: Gentamicin K: Kanamycin, NA: Nalidixic acid, R: Rifampin, S: Streptomycin, TE: Tetracycline

As indicated in Fig. 1, there was a varying degree of resistance in which; 100% resistance was observed for ampicillin, cephalothin and rifampin and on the other hand 100% susceptibility was observed for chloramphenicol, ciprofloxacin, gentamicin, nalidixic acid, kanamycin and tetracycline. Concerning streptomycin, 63.15% of the isolates were susceptible and 36.8% were intermediate. All isolates showed the presence of MDR referring to the resistance of a single isolate against more than two drugs.

## Discussion

In Ethiopia, *E. coli* O157:H7 is considered to be an important challenge for the dairy industry and public health development [47]. This study also indicates *E. coli* O157:H7 to be the major dairy development challenge in the study area. The overall prevalence of *E. coli* O157:H7 was 4.7%. The result is in line with the result reported by Haile et al. [35] who reported a prevalence of 3.5% in food of animal origin in Addis Ababa Ethiopia. This result also supports previous studies [9, 59–62] that reported cattle to be asymptomatic carriers and major reservoirs of *E. coli* serotype O157:H7.

Raw milk can be a vehicle of transmission for *E. coli* O157:H7 [63] and the risk of consumption of raw milk is high; even though the prevalence detected is relatively low [64]. The isolation rate of *E. coli* O157:H7 from raw milk samples were similar to that recorded in the current study (4.54%) was slightly in agreement with the report of 2.9% by Disassa et al. [48]. But, the prevalence is far lower when compared to the reports of Abebe et al. [34] who reported 10.4% from Tigray, Ethiopia. The variations could be because of the differences in animal management, milking systems, and milk hygiene and handling practices from farm to farm and the location of study areas. In the previous studies milking system, milk hygiene and handling practices are more modernized because the farms are intensified with high labor and capital that minimize contact of one milker to many cows, and access to water and other equipment. The detection of *E. coli* O157:H7 from milk is not only a reliable indicator of fecal contamination but also an indicator of poor hygiene and sanitary conditions during milking and handling.

In the present study, a 3.9% isolation rate of *E. coli* O157:H7 was recorded from feces samples. This is in agreement with the prevalence reported by Atinafie et al. [41] and Mersha et al. [45] both reported a prevalence of 4.7% in Hawasa and Modjo, respectively. Isolation of *E. coli* O157:H7 from feces is regarded as important epidemiological information. Inhabited cattle could shed 10^1^ to 10^7^ CFU of *E. coli* O157:H7 per gram of feces. Given that typical cattle excrete 20 to 50 kg of feces per day, this provides large inoculums of *E. coli* O157:H7 for the farm environment and could contaminate dairy products in the presence of poor hygienic practices [65].

Animal drinking water was also identified as one source of *E. coli* O157:H7 in dairy farms [36]. In this study, a prevalence of 8% was reported from water samples. The presence of *E. coli* O157:H7 in water samples may contribute to the prevalence of infection in cattle, a factor directly related to the contamination of dairy products and the environment. Contaminated water can serve as a vehicle for *E. coli* O157:H7 transmission in cattle, although there was variation among animals in the doses necessary to initiate shedding [36, 66].

From the manure samples, a prevalence of 4% was recorded. Farm manure may disseminate, transmit, or propagate *E. coli* O157:H7 and it could be a good vehicle of *E. coli* O157:H7 [59]. Manure sewages from cattle houses could result in contamination of the surrounding land, with cattle keepers and their household members being at increased risk. The survival of *E. coli* O157:H7 in manure depends on many variables, including the level of pathogen shedding by animals, conditions, and duration of manure storage, extraneous microbial interactions within stored manure, and interactions with water [67]. Several researchers have investigated the survival of *E. coli* O157:H7 in manure from various animals, under different conditions such as temperature or aeration, presence of different manure amendments and at a range of manure-to-soil ratios [68]. Kudva et al. [24] found that *E. coli* O157:H7 survived for more than 21 months in ovine manure at levels ranging up to 10^6^ CFU/g manure. Experiments with artificially inoculated bovine feces have also confirmed the survival of *E. coli* O157:H7 for greater than 40 days, dependent on initial inoculums and holding temperature [69].

Many factors were tested for associations with *E. coli* O157:H7, yet relatively few were significant in the final model. Factors such as area (urban, rural), floor type, cleaning of pens, milking location and hand washing during the time of milking were found to be significantly associated with the occurrence of *E. coli* O157:H7. *E. coli* O157:H7 shedding in cattle and its survival in the environment could be multifactorial. No single factor could stand out as the major risk factor for shedding. But, factors related to poor hygienic practices were found to affect the occurrence of the bacteria [63]. It is important to note that, the use of towels and detergents were not significantly associated with the occurrence of *E. coli* O157:H7. This suggests that the use of towels and detergent alone is unlikely to prevent the presence of *E. coli* O157:H7 while the other hygienic practices are poorly practiced. Thus, general hygienic practices might represent a critical control point for reducing the transmission of *E. coli* O157:H7 in dairy farms [49].

In this study, 100% MDR was observed. All isolates were resistant to ampicillin, cephalothin and rifampin. This is in agreement with the report of Bekele et al. [43] and Atinafie [41]. Multidrug resistance occurred due to the misuse of antimicrobial agents or due to genetic mutation [70]. On contrary, all isolates were susceptible to the most commonly used antimicrobials including chloramphenicol, ciprofloxacin, gentamicin [71] and tetracycline. However, Hiko et al. [31], Bekele et al. [43] and Haile et al. [35] were reported resistance to tetracycline which is the most commonly used antimicrobials in Ethiopia, which is contrary to the present study. But, Mohammed et al. [16] reported susceptibility to tetracycline which is in line with the present study.

This study has limitation on the size of a sample that might affect exactness of estimates and it minimizes the power of conclusion given from this study. There is also resource limitation in characterizing and identifying antimicrobial resistance and resistance genes in detail; because lack of materials and equipment needed to perform their procedures such as PCR, DNA microarray, etc.

## Conclusion

The current study revealed a substantial occurrence of *E. coli* O157:H7 in lactating cows and dairy farm environments in ATJK district. *E. coli* O157:H7 was isolated from feces, manure, milk and water designating a sustaining transmission of the bacteria. The occurrence of *E. coli* O157:H7 in milk samples suggests a potential zoonotic risk of raw milk consumption in the area. Factors related to poor hygienic practices such as cleaning of pens, milking location and hand washing were the main factors that backed the occurrence of *E. coli*O157:H7 in the dairy farms. *E. coli* O157:H7 isolates manifested a MDR; 100% resistance to ampicillin, cephalothin and rifampin was observed. Antimicrobials such as chloramphenicol, ciprofloxacin, gentamicin, nalidixic acid, kanamycin and tetracycline may be used as a means to reduce these strains in dairy cattle in ATJK. Comprehensive training should be given to farm owners, milkers and other personnel involved in dairying activity to improve the hygienic practices. Strict animal and environment level hygienic practices should be practiced to break a sustained transmission of the bacteria.

## Supplementary Information

**Additional file 1.**

**Additional file 2.**

## Data Availability

All relevant data are within the paper. Raw data are available with the corresponding author upon request.
