# Supplementary material for: Occurrence of Escherichia Coli O157:H7 in lactating cows and dairy farm environment and the antimicrobial susceptibility pattern at Adami Tulu Jido Kombolcha District, Ethiopia
Source: BMC Vet Res. 2023 Jan 11;19:6. doi: 10.1186/s12917-023-03565-9 (PMC9832739; doi:10.1186/s12917-023-03565-9)
Supplement: Supplementary file 1 — Additional file 1. [file 12917_2023_3565_MOESM1_ESM.pdf]

Annex 1: Bacteriological Medias used for isolation, identification and antimicrobial susceptibility test of *E. coli* O157:H7

Nutrient Agar

Nutrient agar (OXOID® Ltd., Basingstoke, U.K.) containing 1 g/l of '*lab-lecmo'* powder, 2 g/l of yeast extract, 5 g/l of peptone, 5 g/l of sodium chloride and 15 g/l of agar will be prepared according to the manufacturer's instructions. Briefly, 28 g of the powder will be dissolved in 1 liter of distilled water. The solution will be boiled to dissolve completely and sterilized by autoclaving at 121 ºC for 15 minutes. Before use, the media will be cooled up to 45 ºC.

Buffered Peptone Water (BPW)

The medium (BPW powder, Oxoid® Ltd., Basingstoke, Hampshire, England, CM0509, Lot 1442805) is composed of 10 g/l Peptone, 5 g/l Sodium chloride, 3.5 g/l Di-sodium phosphate and 1.5 g/l Potassium di-hydrogen phosphate. The medium will be prepared according to manufacturer's instructions whereby 20 g of the powdered medium will be dissolved in 1 liter of distilled water. The culture medium is mixed well and each 10 ml will be dispensed into capped test tubes. Then, the test tubes are sterilized by autoclaving at 121°C for 15 minutes and cooled to 25°C before use. All the unused prepared media will be stored under refrigeration temperature.

Normal saline solution

The solution will be prepared by dissolving 0.85 g of Sodium chloride (Sigma-Aldrich, Co., USA, and Cat. S5886, Lot SLBC3215V) into 100 ml of sterile distilled water, mixed well and sterilizing by autoclaving at 121°C for 15 minutes and cooled to below 45°C, the solution will be ready for use.

Mueller-Hinton (MH) Agar

The medium (Oxoid® Ltd., Basingstoke, Hampshire, England, CM0337 Lot 744451) is composed of 300 g/l Beef, dehydrated infusion, 17.5 g/l Casein hydrolysate, 1.5 g/l Starch, 17 g/l Agar and final pH of 7.3±0.1 at 25°C. The medium will be prepared according to manufacturer's instructions whereby 38 g of the powdered medium is suspended into 1 liter of distilled water, mixed well and brought to boil to dissolve the medium completely. Then, the medium is sterilized by autoclaving at 121°C for 15 minutes, cooled to below 45°C and poured into sterile

Petri dishes. The plates are left at room temperature for two hours for the media to solidify then put upside down in the incubator for 24 hours at 37°C to check for sterility and to dry the condensed vapour on the plate cover.

Sorbitol MacConkey (SMAC) Agar

Sorbitol MacConkey (SMAC) Agar (Oxoid®, Ltd., Basingstoke, Hampshire, England, CM0813 and Lot 1116827) is composed of Peptone (10 g/l), Sorbitol (10 g/l), bile salts No.3 (1.5 g/l), Sodium chloride (5g/l), Neutral red (0.03 g/l), crystal violet (0.001 g/l) Agar (15 g/l) and final pH of 7.1±0.2 at 25°C. It will be prepared according to manufacturer's instruction, whereby 51.5g powder medium will be suspended in one liter of distilled water and brought to the boil to dissolve completely. Then it will be sterilized by autoclaving at 121°C for 15 minutes. Thereafter it will be allowed to cool to 50°C and poured into sterile Petri dishes, and lastly allowed to solidify at room temperature, and stored upside down at 4 to 8°C, refrigerator, for subsequent use.

Methyl Red and Voges-Proskauer (MR-VP) medium

MR-VP Medium (Himedia Laboratories Pvt, Ltd., Mumbai-400086, India, M070I Lot 0000219697) is composed of Buffered peptone (7g/l), Dextrose (5 g/l), Dipotassium phosphate (5g/l) and final pH 6.9±0.2 at 25°C. The medium will be prepared according to manufacturer's instruction by which 17 grams of powder will be suspended in one liter of distilled water. The medium will be heated (if necessary) to dissolve it completely and then sterilized by autoclaving 121°C for 15 minutes. Then it will be allowed to cool and two sets of test tubes were dispended by MR-VP broth (3 mL, Voges-Proskauer and 5 mL, Methyl Red). The tubes will be placed in refrigerator if not meanwhile the inoculum is prepared.

Simmons citrate agar

The medium (Himedia Laboratories Pvt, Ltd., Mumbai-400086, India, M099, Lot 0000163279) is composed of Magnesium Sulfate (heptahydrate) (0.2 g/l), Ammonium Dihydrogen Phosphate (1 g/l), Dipotassium Phosphate (1 g/l), Sodium Citrate (dehydrate) (2 g/l), Sodium Chloride (NaCl) (0.08 g/l), Bromothymol blue (0.08 g/l) and Agar (15 g/l) adjusted at final pH of 6.8±0.2 at 25°C. It will be prepared according to manufacturer's instruction by which 24.28 gram of

powder will be suspended in one liter of distilled water. The medium will be heated to boil to dissolve the medium completely and then sterilized by autoclaving 121°C for 15 minutes. Then after it will be cooled to 45-50^{0}C and poured in to sterile test tubes, in so doing cooled in slanted position and finally stored in a refrigerator to ensure a shelf life.

Wash buffer: modified phosphate buffer

The medium is composed of sodium chloride (8g), potassium chloride (0.2g), Disodium hydrogen phosphate (1.44g), potassium dihydrogen phosphate (0.24g) and polyoxylethylene sorbitan monnolaurate (0.2g) adjusted at final pH of 7.3±0.1 at 25^{0}C. It will be prepared according to manufacturer's instruction and the medium will be heated to boil to dissolve the medium completely and then sterilized by autoclaving 121°C for 15 minutes.
