# Supplementary material for: Occurrence of Escherichia Coli O157:H7 in lactating cows and dairy farm environment and the antimicrobial susceptibility pattern at Adami Tulu Jido Kombolcha District, Ethiopia
Source: BMC Vet Res. 2023 Jan 11;19:6. doi: 10.1186/s12917-023-03565-9 (PMC9832739; doi:10.1186/s12917-023-03565-9)
Supplement: Supplementary file 2 — Additional file 2. [file 12917_2023_3565_MOESM2_ESM.pdf]

Questionnaire format

Format 1: Questionnaire format for farm owner

Farm ID or No: ____ Date_______

1. Farm name_______________________ Address___________

2. Scale and type of dairy farm: ________ Government______________Private____________

3. Herd size________ Breed: local_________ cross ________ exotic_______

5. Feed and water hygiene and storage: Excellent ☐ very good ☐ Good ☐ Fair ☐ Poor ☐

6. If your animals are enclosed, what type of animal house floor is in? (Only single choice)

Covered with manure ☐ Concrete ☐ Earthed floor ☐ Others (specify)________

7. How often the barn and/or the milking room are/is cleaned?

Twice a day ☐ Once a day ☐ Once per two days ☐ Others (sepecify)_____

8. Where do cows milked? In barn ☐ In milking room ☐ Any where ☐

9. How do you milk your cows? By hand milking ☐ By milking machine ☐

10. Milking frequency per day: Once ☐ Twice ☐ Three times ☐ If more label_____

11. When do you wash your hands? (Encircle)

a. Before and after milking
b. Between milking
c. Only before milking
d. Only after milking
e. Not at all

12. When do you use teat bathe and towel? (Encircle)

a. Before milking
b. After milking
c. Between milking
d. before and after milking
e. Don’t use any dip and towel

13. Do you sell raw milk to customers? Every day ☐ Sometimes ☐ Never ☐

13.1. When you sell? Every morning ☐ Afternoon ☐ Evening ☐

13.2. Have you transportation facility to deliver milk for your customer?

13.3. Where does the milk go? To household consumption____ To restaurants____ To___

14. How do you keep milk containers and milking buckets? Washing with:

a. Warm water
b. Cold water
c. Both warm and cold water
d. Detergents

15. Milker's clothing: Boot______Clean outer garment________ Ordinary own cloth__________Apron______ others (specify) ___________ Interviewer observation_____________

16. Do you sell raw milk to customers? Every day ☐ Sometimes ☐ Never ☐

16.1. When you sell? Every morning ☐ Afternoon ☐ Evening ☐

16.2. Have you transportation facility to deliver milk for your customer? Yes ☐ No ☐

16.3. Where does the milk go?

17. Do lactating herd experience mastitis at the farm? Yes__________ No__________________

17.1. If your answer is yes, who treated mastitis cow? ________________________________

18. Is there any practice of record keeping? Yes____________ No_________________________

If yes: Breeding records_________ Calving records_______ Production records________ Health records________ Financial records____________ Feeding records_______________ Others_______
